# Supplementary material for: CPT2 Deficiency Modeled in Zebrafish: Abnormal Neural Development, Electrical Activity, Behavior, and Schizophrenia-Related Gene Expression
Source: Biomolecules. 2024 Jul 26;14(8):914. doi: 10.3390/biom14080914 (PMC11353230; doi:10.3390/biom14080914)
Supplement: Supplementary file 1 [file biomolecules-14-00914-s001.zip › biomolecules-3086972-supplementary.pdf]

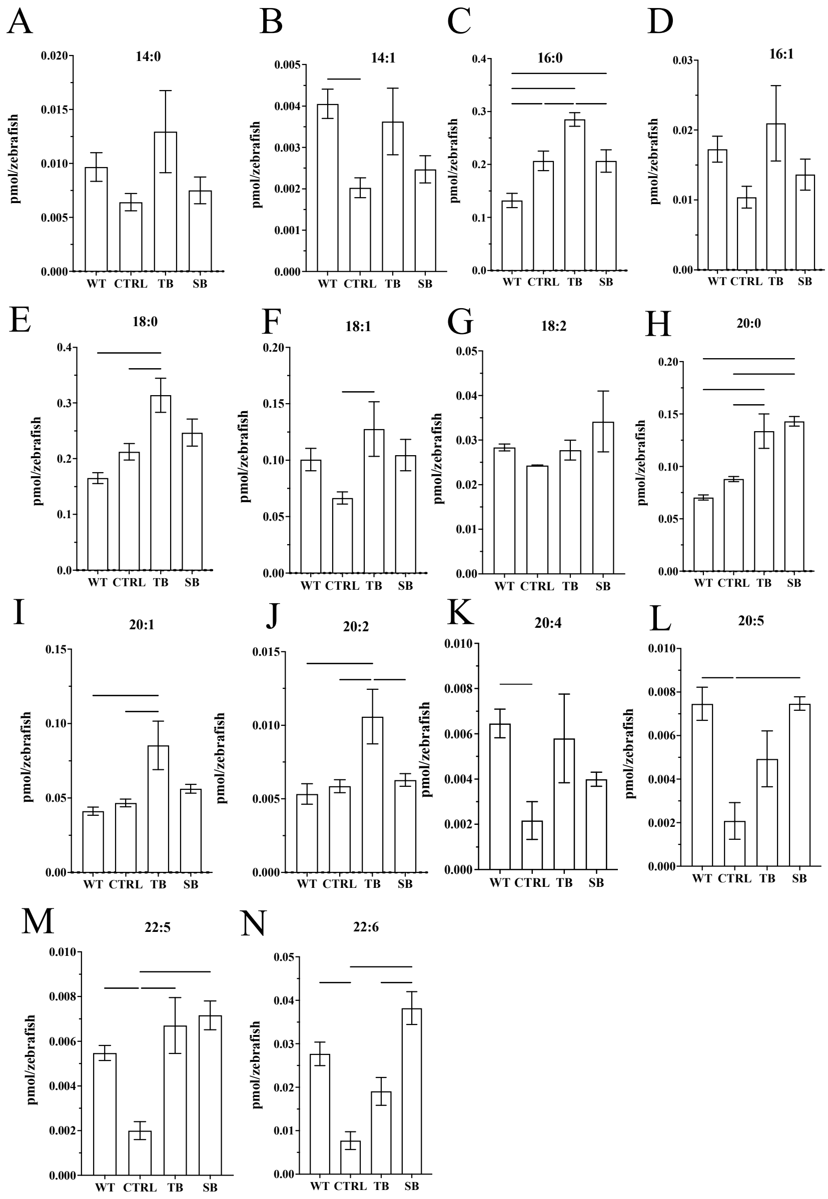

Figure S1. *LC-MS/MS Analysis for Fatty-Acylcarnitine Species.* **A-N.** Acylcarnitine species expression in CPT2 knockdown larvae. WT=wildtype, CTRL= control MO, TB=translation blocking MO, SB=splice blocking MO. N ≤ 30 fish per condition for each of five trials. *p ≤ 0.05, **p < 0.01, ***p < 0.001, ****p < 0.0001. Error bars = SEM.

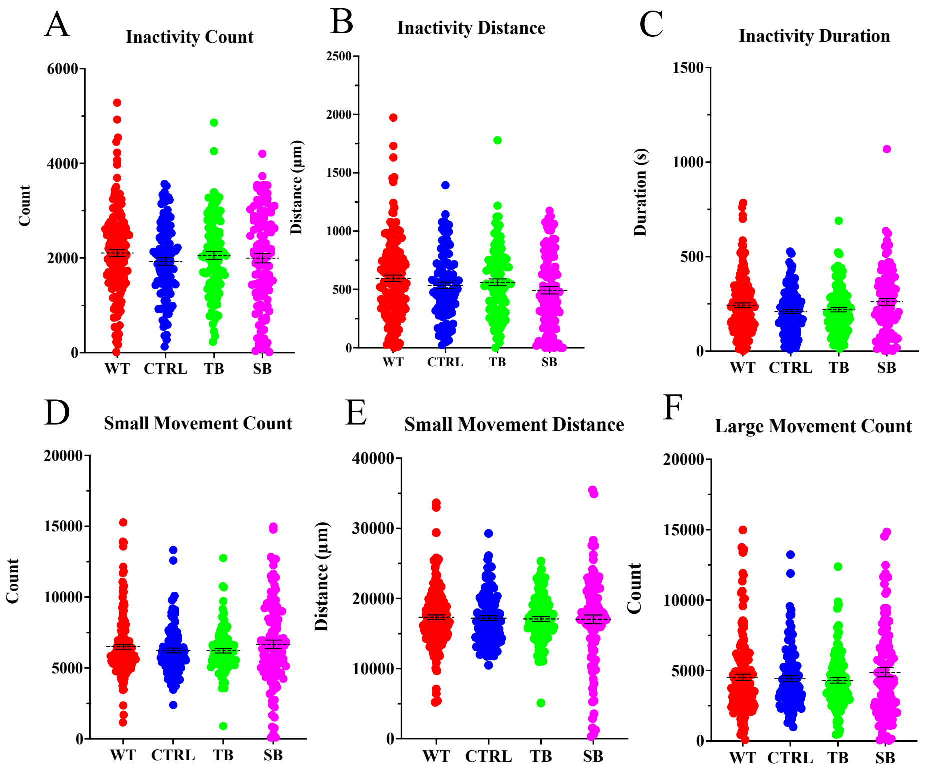

Figure S2. *Tracking Behavior Assay of Zebrafish Swimming Behavior.* **A-F.** Tracking of zebrafish swimming movement count, distance, and duration. WT = wildtype, CTRL = control MO, TB = translation blocking MO, SB= splice blocking MO. N= 60 fish used per condition for each of two trials. Error bars = SEM.

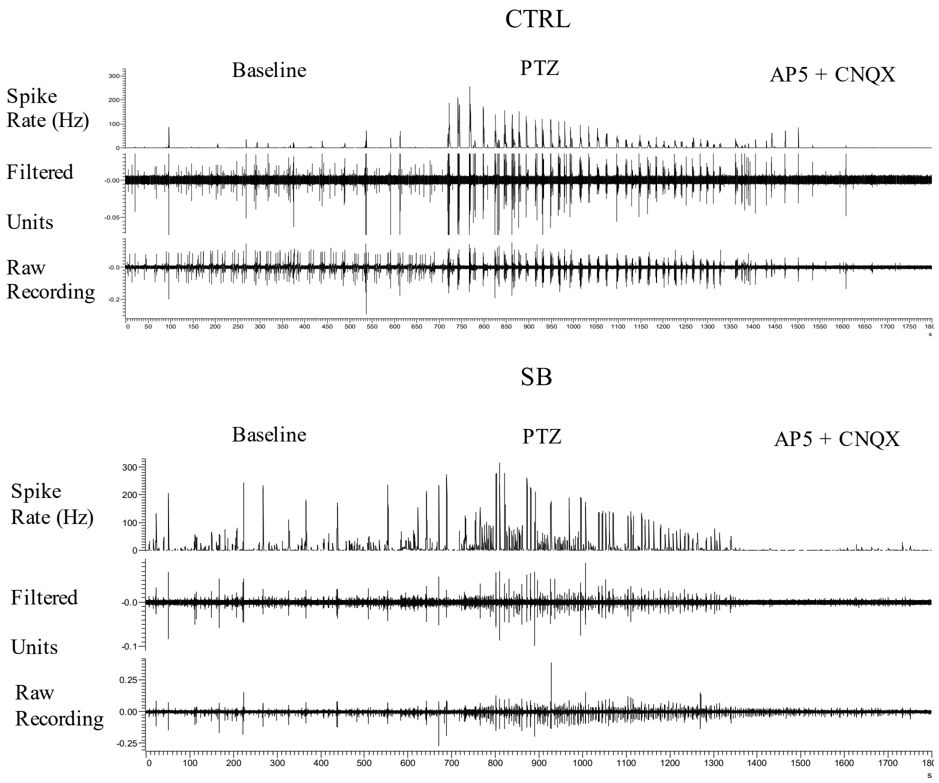

Figure S3. *Seizure Assay Recording Traces.* Whole 30 min recording trace through aCSF, PTZ, and AP5 + CNQX perfusion. Raw recording, filtered multi-units recording, and spike rate were determined for CTRL and SB larvae.
